# A synthetic cationic antimicrobial peptide inhibits inflammatory response and the NLRP3 inflammasome by neutralizing LPS and ATP

**DOI:** 10.1371/journal.pone.0182057

**Published:** 2017-07-27

**Authors:** Lan-Hui Li, Tz-Chuen Ju, Chih-Yu Hsieh, Wei-Chih Dong, Wan-Tze Chen, Kuo-Feng Hua, Wei-Jung Chen

**Affiliations:** 1 Department of Laboratory Medicine, Lisen, Chinese Medicine and Kunming Branch, Taipei City Hospital, Taipei, Taiwan; 2 Department of Nursing, St. Mary's Junior College of Medicine, Nursing and Management, Ilan, Taiwan; 3 Department of Biotechnology and Animal Science, National Ilan University, Ilan, Taiwan; 4 Department of Pathology, Tri-Service General Hospital, National Defense Medical Center, Taipei, Taiwan; National Institutes of Health, UNITED STATES

## Abstract

Antimicrobial peptides (AMPs) are one of the most important defense mechanisms against bacterial infections in insects, plants, non-mammalian vertebrates, and mammals. In the present study, a class of synthetic AMPs was evaluated for anti-inflammatory activity. One cationic AMP, GW-A2, demonstrated the ability to inhibit the expression levels of nitric oxide (NO), inducible NO synthase (iNOS), cyclooxygenase-2 (COX-2), tumor necrosis factor-α (TNF-α) and interleukin-6 (IL-6) in lipopolysaccharide (LPS)-activated macrophages. GW-A2 reduced LPS-induced increases in the phosphorylation of mitogen-activated protein kinase and protein kinase C-α/δ and the activation of NF-κB. GW-A2 also inhibited NLRP3 inflammasome activation induced by LPS and ATP. Furthermore, in the mice injected with LPS, GW-A2 reduced (1) the concentration of IL-1β, IL-6 and TNF-α in the serum; (2) the concentration of TNF-α in the peritoneal lavage; (3) the expression levels of iNOS, COX-2 and NLRP3 in the liver and lung; (4) the infiltration of polymorphonuclear neutrophils in the liver and lung. The underlying mechanisms for the anti-inflammatory activity of GW-A2 were found to be partially due to LPS and ATP neutralization. These results provide insights into how GW-A2 inhibits inflammation and the NLRP3 inflammasome and provide a foundation for the design of rational therapeutics for inflammation-related diseases.

## Introduction

Expression of cationic host defense peptides (also known as antimicrobial peptides, or AMPs) is a mechanism used by the host to defend against pathogenic microbes. This mechanism is present in plants, insects, non-mammalian vertebrates, and mammals [1]. AMPs are recognized as a potential therapeutic strategy against infection [2–5]. In addition to their antimicrobial activities, AMPs possess medicinal and pharmacological activities, such as anti-cancer [6,7] and immunomodulatory capabilities in both pro- and anti-inflammatory responses [8,9]. Recently, it has been reported that AMPs play important roles in disease development. For example, cathelicidin-related AMP, the mouse homolog of human LL-37, induces regulatory immune cells in pancreatic islets and limits autoimmune diabetes [10]. Elevated plasma levels of cathelicidin-related AMP were found in patients with liver disease, and it can also have a protective effect toward mouse liver injury [11]. Additionally, Lee et al. showed that human AMP LL-37 plays a role in chronic neuroinflammation in Alzheimer's and Parkinson's disease by inducing inflammatory cytokine secretion by microglia, astrocytes, THP-1 cells and U373 cells [12].

A short-lived and well-regulated inflammatory response helps fight infection; however, a prolonged and uncontrolled response, characterized by excessive cytokine production, can be harmful because it can cause host toxicity and tissue damage [13]. Lipopolysaccharide (LPS) is a negatively charged cell wall component of Gram-negative bacteria. The binding of Toll-like receptor 4 by LPS initiates an inflammatory response [14]. Interleukin-1β (IL-1β) is an important activator of inflammation because it amplifies inflammatory responses by inducing the cytokines TNF-α and IL-6 [15,16]. The NLRP3 inflammasome controls IL-1β secretion. The NLRP3 inflammasome is a caspase-1-containing protein complex that cleaves immature IL-1β precursor (proIL-1β) into mature IL-1β [17]. Full activation of the NLRP3 inflammasome requires both a priming signal from LPS and an activation signal from ATP; the former controls the expression of NLRP3 and proIL-1β and the latter controls caspase-1 activation [17]. The NLRP3 inflammasome controls the pathogeneses of many important diseases, such as type II diabetes [18], Alzheimer's disease [19], atherosclerosis [20] and gout [21]. Although AMPs have been reported to inhibit LPS-mediated inflammation in vitro and in vivo [22,23], the effects of AMPs on NLRP3 inflammasome activation is unclear [24].

Recently, we synthesized a class of cationic AMPs and studied their anti-bacterial activities, as well as their interactions with bacteria [25–29]. We showed that these synthetic AMPs exhibit high cytotoxicity against various cancer cells by inducing apoptosis [30–32]. In the current study, a class of synthetic AMPs was evaluated for anti-inflammatory activity in vitro and in vivo. The underlying mechanisms of the anti-inflammatory activity involved neutralization of LPS and ATP, which resulted in reduced cytokine production and NLRP3 inflammasome activation.

## Materials and methods

### Materials

LPS from *Escherichia coli* 0111:B4 (L2630), LPS from *Salmonella enterica* serotype minnesota Re 595 (L9764), peptidoglycan (79682), and actin antibody (A2228) were purchased from Sigma (St. Louis, MO, USA). Antibodies for phospho-PKC-α (sc-12356), phospho-PKC-δ (sc-11770), PKC (sc-80), phospho-IκB-α (sc-8404), IκB-α (sc-371), iNOS (sc-651), COX-2 (sc-1745), ERK1/2 (sc-135990), JNK1 (sc-1648) and p38α (sc-535) antibodies were obtained from Santa Cruz Biotechnology (Santa Cruz, CA, USA). Antibody for NLRP3 (AG-20B-0006-C100) was purchased from Adipogen (San Diego, CA, USA). Mouse IL-1β (88-7013-88), mouse IL-6 (88-7064-88), mouse TNF-α (88-7324-88), human IL-6 (88-7066-88) and human TNF-α (88-7346-88) ELISA kits were purchased from eBioscience (San Diego, CA, USA). MAPK family antibody sampler kit (9910T) was purchased from Cell Signaling Technology, Inc (Danvers, MA, USA). Pam3CSK4 (tlrl-pms), ATP (tlrl-atp), nigericin (tlrl-nig) and monosodium urate crystal (MSU) (tlrl-msu) were purchased from InvivoGen (San Diego, CA, USA).

### AMPs

AMPs were designed based on four structural determinants: charge (Q), polar angle (θ), hydrophobicity (H), and hydrophobic moment (M_H_), as described in our previous work [25]. Two naturally occurring and potent AMPs, pleurocidin (from winter flounder) and magainin 2a (from frog), were compared against the synthetic AMPs. The amino acid sequences, structural parameters, and molecular masses determined for the 16 AMPs used in the current study are summarized in Table 1. Peptides were synthesized on a solid-phase peptide synthesizer (Model 433A; Applied Biosystems), as previously described [25]. All peptides were purified by RP-HPLC to a final purity of >95%, followed by MALDI-TOF mass spectrometry analysis.

**Table 1:** Characterization of synthetic and natural AMPs and their effects on cell viability and LPS-induced NO generation in RAW 264.7 cells.

Group ^a^	Peptide	Amino Acid Sequence	Parameters				*M*_r_		LC_50_ ^c^	ED_50_ ^d^
			Q	θ	H	M_H_	*Estimated*	*Experimental*	(μM)	(μM)
**Q**	**GW-Q3**	GANLAKKFYTYINKFINYAW	+3	140^o^	-0.043	0.343	2425.81	2426.00	>8	7
	**GW-Q4**	GANAA**KK**FATIA**KK**FINYLW	+4	140 ^o^	-0.043	0.344	2255.69	2256.26	>8	>8
	**GW-Q5**	GANAL**KK**YFTIL**KK**FF**K**LAW	+5	140 ^o^	-0.044	0.343	2387.94	2388.80	8	2
	**GW-Q6**	GIKIA**KK**AITIA**KK**IA**K**IYW	+6	140 ^o^	-0.044	0.343	2257.88	2258.00	>8	4
**θ**	**GW-A1**	GA**K**YA**K**YIYNFY**K**YIA**K**YIW	+4	100 ^o^	-0.042	0.343	2567.03	2567.60	>8	>8
	**GW-A2**	GA**K**YA**K**IIYNYL**KK**IANALW	+4	120 ^o^	-0.042	0.344	2341.82	2341.60	11	2
	**GW-A4**	GA**K**ALT**K**AATAFT**K**FY**K**TIW	+4	160 ^o^	-0.042	0.345	2217.64	2218.40	>8	4
	**GW-A5**	GATYAKKIIKTITKIATTAW	+4	180 ^o^	-0.042	0.344	2179.63	2180.54	>8	>8
**H**	**GW-H0**	GY**K**YYN**K**IYNYLN**K**YL**K**YAW	+4	140 ^o^	-0.181	0.345	2669.08	2669.01	>8	5
	**GW-H1**	GYNYA**KK**LANLA**KK**FANALW	+4	140 ^o^	-0.115	0.344	2284.69	2284.80	>8	>8
	**GW-H3**	GLTFL**KK**ILNFA**KK**IYTAIW	+4	140 ^o^	0.033	0.343	2368.93	2368.80	>8	4
**M**_**H**_	**GW-M1**	GANAA**KK**LATFA**KK**IFTAYW	+4	140 ^o^	-0.044	0.291	2200.61	2200.66	>8	4
	**GW-M3**	GANAA**KK**FANLI**KK**IFNYIW	+4	140 ^o^	-0.042	0.400	2310.77	2310.94	>8	2
	**GW-M4**	GY**K**YINNII**K**YIN**K**FF**K**YIW	+4	140 ^o^	-0.042	0.451	2629.14	2629.06	7	2
**Natural**	**M2a**	GIG**K**FLHSA**KK**WG**K**AFVGEIMNS	+4	140 ^o^	-0.046	0.325	2505.96	2506.26	5	>8
**AMPs** ^**b**^	**Ple**	GWGSFF**KK**AAHVG**K**HVG**K**AALTHYL	+4	140 ^o^	-0.026	0.287	2711.17	2711.46	>8	>8

^a^ AMPs were designed based on four major structural parameters: charge (Q), polar angle (**θ**), hydrophobicity (H), and hydrophobic moment (M_H_).

^b^ M2a, Magainin 2a from frog. Ple, Pleurocidin from winter flounder.

^c^ LC_50_, lethal concentration of AMPs that kills 50% of RAW264.7 cells. The data are expressed as the mean values from triplicate wells from at least three experiments.

^d^ ED_50_, effective dose of AMPs that inhibits 50% of LPS-induced NO generation in RAW264.7 cells. The data are expressed as the mean values from triplicate wells from at least three experiments.

### Cell cultures

RAW-Blue™ cells were purchased from InvivoGen (San Diego, CA) and the other cell lines used in this study were obtained from American Type Culture Collection (Rockville, MD, USA). All cells were propagated in RPMI-1640 medium as described in detail previously [33]. To induce monocyte-to-macrophage differentiation, THP-1 cells were cultured for 48 h in RPMI-1640 medium supplemented with 100 nM phorbol 12-myristate 13-acetate (P1585, Sigma).

### Cell viability assay

RAW 264.7 cells or THP-1 macrophages (5 × 10^3^ in 0.1 ml of medium) were seeded in 96-well plates and then incubated with various doses of AMP for 24 h. An AlamarBlue® assay (AbD Serotec, Oxford, UK) was used to determine the cytotoxicity of the test AMP. The procedure was conducted following the protocol described in the manufacturer’s instructions.

### NO inhibitory assay

RAW 264.7 cells (2 × 10^5^ in 0.5 ml of medium) were seeded in 24-well plates and then incubated with or without LPS (0.1 μg/ml) in the absence or presence of AMP for 24 h. The effects of AMP on NO production were measured indirectly by analyzing nitrite levels using the Griess reaction as described in detail previously [33].

### Enzyme-linked immunosorbent assay (ELISA)

The expression levels of IL-1β, IL-6 and TNF-α in the conditional medium, sera and lavage fluids were measured by ELISA as described in detail previously [33].

### Western blot assay

The protein expression levels of iNOS, COX-2, ERK1/2, JNK1/2, p38, IκB-α, PKC, caspase-1 and the phosphorylation levels of ERK1/2, JNK1/2, p38, PKC-α, PKC-δ, IκB-α were determined by Western blotting as described in detail previously [33].

### NF-κB reporter assay

RAW-Blue™ cells (2 × 10^5^ in 0.5 ml of medium) were seeded in 24-well plates and then incubated with LPS (0.1 μg/ml) in the presence or absence of AMPs for 24 h. The NF-κB activity was assayed by QUANTI-Blue™ (rep-qb1, InvivoGen) as described in detail previously [33].

### Flow cytometry

For binding of GW-A2 to the cell surface, RAW 264.7 cells were fixed for 15 min with 2% paraformaldehyde and then incubated for 30 min with 20 μM of GW-A2-FITC at 4°C. After washing, cells were subjected to flow cytometric analysis on a FACSCalibur using CellQuest Software (Becton Dickinson Inc., San Jose, CA, USA). To assess binding of LPS to the cell surface, RAW 264.7 cells were fixed for 15 min with 2% paraformaldehyde and then incubated for 30 min with GW-A2. This was followed by incubation for 30 min with 2 μg/ml of LPS-FITC **(**F3665, Sigma**)** at 4°C. After washing, the cells were subjected to flow cytometric analysis.

### Quantitative real-time PCR

RAW 264.7 (2 × 10^6^ in 2 ml of medium) were seeded in 6-cm dishes and then treated with or without LPS (0.1 μg/ml) in the absence or presence of AMP for 6 h. Total RNA was extracted from the treated cells by TRIzol reagent (Gibco, Grand Island, NY, USA) and the cDNA was prepared as described in detail previously [34]. The mRNA expression levels of iNOS and COX-2 were measured by quantitative real-time PCR using the StepOne real-time PCR system (Applied Biosystems, Foster City, CA, USA). The primers used were: for mouse iNOS, forward 5- GGAGCCTTTAGACCTCAACAGA-3 and reverse 5-TGAACGAGGAGGGTGGTG-3; for mouse COX-2, forward 5-CACTACATCCTGACCCACTT-3 and reverse 5-ATGCTCCTGCTTGAGTATGT-3. The gene expression data are presented as the relative expression (LPS group as 100%) normalized to that of glyceraldehyde-3-phosphate dehydrogenase (GAPDH).

### ImmunoMagnetic Reduction (IMR) assay

The methodology of IMR assay was described in detail previously [35]. Briefly, magnetic particles, with a mean diameter of 53.8 nm and a dextran and GW-A2 coating, were dispersed in PBS solution (pH = 7.2). GW-A2 was covalently bound onto dextran to obtain magnetic reagents for detecting LPS and ATP. The magnetic concentration of the reagent was 0.1 emu/g. The magnetic reagent was stored at 4°C. A XacPro-E (MagQu Co., Ltd.) was used to detect reagent IMR signals, which were released by the association between magnetic particles and detected LPS and ATP. The LPS and ATP solutions were diluted with pH 7.2 PBS to final concentrations of 0.1 to 10,000 ng/ml and 0.1 to 1000 ng/ml, respectively. One each of the LPS and ATP dilutions were used for IMR measurement. The others were left at 4°C. The magnetic reagent was transferred from 4°C to room temperature, and it remained at room temperature for 5 min. The magnetic reagent and either the LPS or ATP solution were vortexed for 15 seconds. A mixture of 40 μl magnetic reagent and 60 μl LPS or ATP solution was pipetted into a glass tube. The remaining magnetic reagent and LPS or ATP solutions were stored at 4°C. The mixture of magnetic reagent and the LPS or ATP solution was vortexed for 15 seconds. The mixture was transferred to an XacPro-E to measure IMR signal.

### Ethics statement

All animal manipulations were performed in the Laboratory Animal Center of National Ilan University (Ilan, Taiwan) in accordance with the National Ilan University guide for the care and use of laboratory animals. The procedures used were approved by the Animal Care and Use Committee of National Ilan University. The mice were humanely euthanized by inhalation of an overdose of isoflurane to minimize suffering.

### Animal experiments

Experiments were performed on 8-week-old male C57BL/6 mice purchased from the National Laboratory Animal Center (Taipei, Taiwan). The mice were randomized into the following four groups: Group I: control, intraperitoneal injection of 100 μl of sterile PBS, n = 3; Group II: LPS, intraperitoneal injection of 100 μl of sterile PBS containing *E*. *coli* LPS (10 mg/kg), n = 5; Group III: GW-A2+LPS, intraperitoneal injection of 100 μl of sterile PBS containing *E*. *coli* LPS (10 mg/kg) and GW-A2 (10 mg/kg), n = 5. Group IV: AMP, intraperitoneal injection of 100 μl of sterile PBS containing GW-A2 (10 mg/kg), n = 3. Sera were collected for analysis at 4 h after LPS injection. Peritoneal lavages, lungs and livers were collected for analysis at 24 h after LPS injection.

### Histopathology analysis

Lungs, livers and kidney were fixed in 10% formalin and paraffin-embedded, then 4 um thick sections were cut and stained with hematoxylin and eosin was described in detail previously [36]. Scoring of the polymorphonuclear neutrophils (PMN) infiltration was determined by counting in 5 randomly sampled fields by light microscopy at a magnification of x400 evaluated by a pathologist in National Defense Medical Center (Taipei, Taiwan) [36].

### Statistical analysis

All values represent the mean ± SD. Data were analyzed using t-test.

## Results

### Effects of AMPs on cell viability and NO generation

Sixteen synthetic AMPs were evaluated for cytotoxicity and anti-inflammatory activity in *E*. *coli* LPS-activated RAW264.7 macrophages. The values corresponding to the cytotoxicity and NO inhibitory activity of each tested AMP are shown in Table 1. Among the tested AMPs, GW-Q5, GW-A2, GW-M3 and GW-M4 inhibited NO generation in *E*. *coli* LPS-activated macrophages, with ED_50_ values of approximately 2 μM. GW-A2 showed relatively lower cytotoxicity than the others. Thus, in the present study, the anti-inflammatory activity of and mechanisms underlying the effects produced by GW-A2 were further examined.

### GW-A2 reduces pro-inflammatory mediator expression in LPS-activated macrophages

**Fig 1:**
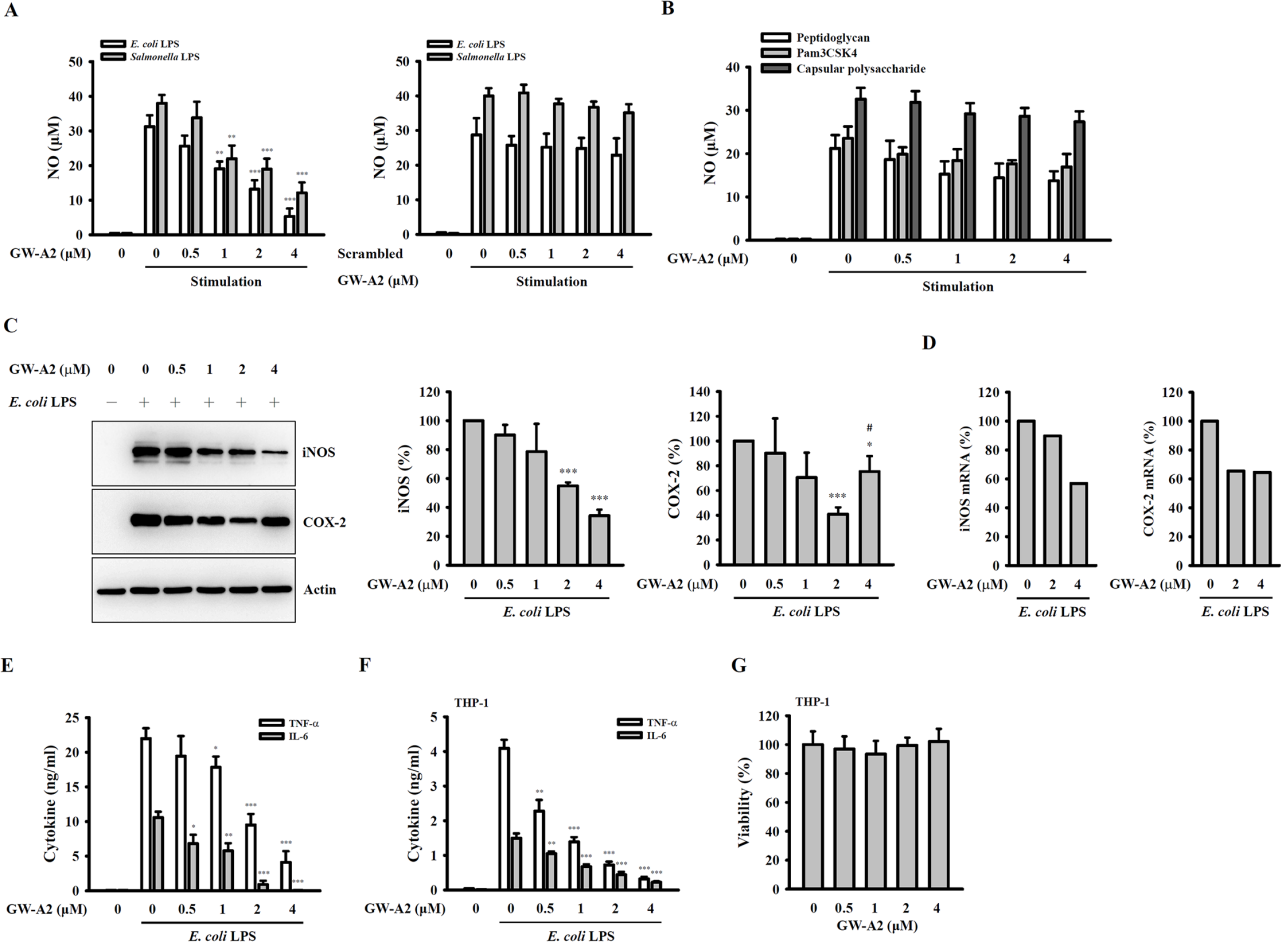
Effects of GW-A2 on the expression levels of inflammatory mediators. (**A**) RAW 264.7 macrophages were incubated for 30 min with or without GW-A2 or scrambled GW-A2, followed by incubation with or without 0.1 μg/ml of *E*. *coli* LPS or *Salmonella* LPS for 24 h. The levels of NO in the culture medium were measured by the Griess reaction. (**B**) RAW 264.7 macrophages were incubated for 30 min with or without GW-A2, followed by incubation with or without peptidoglycan (20 μg/ml), Pam3CSK4 (0.2 μg/ml) or capsular polysaccharide (3 μg/ml) for 24 h. The levels of NO in the culture medium were measured by the Griess reaction. (**C**) RAW 264.7 macrophages were incubated for 30 min with or without GW-A2, followed by incubation with or without 0.1 μg/ml of *E*. *coli* LPS for 24 h. The levels of iNOS and COX-2 were assessed by Western blotting and the histograms represent the expression levels compared to the LPS alone group obtained in three different experiments. (**D**) RAW 264.7 macrophages were incubated for 30 min with or without GW-A2, followed by incubation with or without 0.1 μg/ml of *E*. *coli* LPS for 6 h. The mRNA levels of iNOS and COX-2 were assessed by quantitative real-time PCR. (**E** and **F**) RAW 264.7 macrophages (**E**) and human THP-1 macrophages (**F**) were incubated for 30 min with or without GW-A2, followed by incubation with or without 0.1 μg/ml of *E*. *coli* LPS for 24 h. The levels of TNF-α and IL-6 were assayed by ELISA. (**G**) THP-1 macrophages were incubated for 24 h with or without GW-A2. The cell proliferation was measured by AlamarBlue® assay. The data are expressed as the mean ± SD of three independent experiments. *, ** and *** indicate significant differences, representing *p* < 0.05, *p* < 0.01 and *p* < 0.001, respectively compared to stimulation alone. # indicates significant differences, representing *p* < 0.05 compared to 2 μM of GW-A2 group.

The anti-inflammatory activity of GW-A2 was investigated using LPS-activated RAW 264.7 macrophages. We found that NO generation, which was induced by *E*. *coli*-LPS and *Salmonella*-LPS, was inhibited by GW-A2 in a dose-dependent manner. However, scrambled GW-A2 did not significantly inhibit the NO generation that was induced by *E*. *coli*-LPS and *Salmonella*-LPS (Fig 1A). We further investigated the effects of GW-A2 on NO generation induced by other bacterial components, including PGN from *Staphylococcus aureus*; Pam3CSK4, a synthetic triacylated lipopeptide that mimics the acylated amino acid termini of bacterial lipoproteins; and capsular polysaccharide (CPS) from *Klebsiella pneumoniae* [37,38]. As shown in Fig 1B, the NO inhibitory activities of GW-A2 in PGN-, Pam3CSK4- and CPS-activated macrophages were much lower than that in LPS-activated macrophages. In addition, GW-A2 inhibited the protein (Fig 1C) and mRNA (Fig 1D) expression levels of iNOS and COX-2 in *E*. *coli*-LPS-activated RAW 264.7 macrophages. Interestingly, compared to the 2 μM of GW-A2, 4 μM of GW-A2 had lower inhibitory activity on COX-2 protein expression. However, the detailed mechanism needs further investigation. In addition, GW-A2 also reduced the secretion levels of TNF-α and IL-6 induced by *E*. *coli*-LPS (Fig 1E). The inhibitory effect of GW-A2 on TNF-α and IL-6 secretion was further confirmed by using *E*. *coli*-LPS-activated human THP-1 macrophages (Fig 1F). The reduced cytokine secretion in THP-1 macrophages was not due to cytotoxic effects, as GW-A2 did not reduce the viability of THP-1 macrophages (Fig 1G).

### GW-A2 reduces MAPK and PKC-α/δ phosphorylation and NF-κB activation

**Fig 2:**
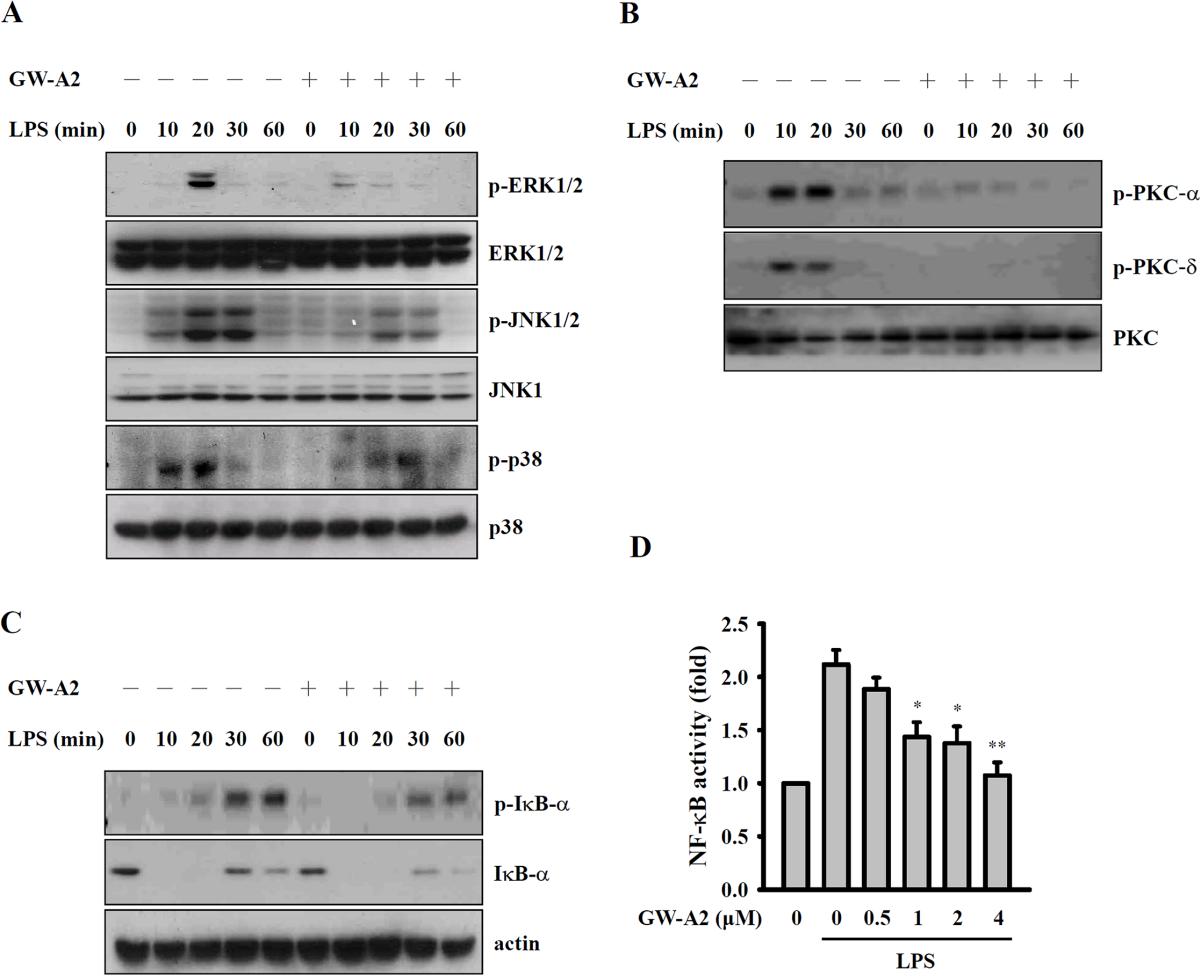
Effects of GW-A2 on LPS-mediated signaling pathways. (**A-C**) RAW 264.7 macrophages were incubated for 30 min with or without 2 μM of GW-A2, followed by 0–60 min with or without 0.1 μg/ml of *E*. *coli* LPS. The phosphorylation levels of (**A**) ERK1/2, JNK1/2 and p38, (**B**) PKC-α and PKC-δ, and (**C**) I-κB-α were assayed by western blotting. (**D**) RAW-Blue^TM^ cells were incubated for 30 min with or without GW-A2, followed by 24 h with or without 0.1 μg/ml of *E*. *coli* LPS. The activation levels of NF-κB were measured by an NF-κB reporter assay. The data are expressed as the mean ± SD of three independent experiments. The western blotting results shown are a representative experiment of three independent experiments. * and ** indicate significant differences, representing *p* < 0.05 and *p* < 0.01, respectively compared to LPS alone.

LPS induces macrophages to produce inflammatory mediators through a variety of signaling pathways, including the MAPK, PKC and NF-κB signaling pathways [39]. In the present study, we found that *E*. *coli*-LPS induces an increase in the phosphorylation levels of ERK1/2, JNK1/2 and p38 and that these effects are reduced by GW-A2 (Fig 2A). In particular, PKC is one of the signaling components of TLR4, and it has roles in macrophage activation in response to LPS [39]. Therefore, we examined the effects of GW-A2 on *E*. *coli*-LPS-induced PKC activation. As shown in Fig 2B, LPS induced an increase in the phosphorylation levels of PKC-α and PKC-δ, and these effects were reduced by GW-A2. In resting macrophages, IκB inhibits NF-κB activity, and NF-κB can be activated by LPS stimulation [40]. Inhibition of activated NF-κB results in a reduction of NO generation and cytokine secretion in LPS-activated macrophages [39]. As shown in Fig 2C, GW-A2 inhibited the phosphorylation levels of IκB-α in *E*. *coli*-LPS-activated macrophages. In addition, using NF-κB-dependent alkaline phosphatase reporter cells, we demonstrated that NF-κB transcriptional activity in *E*. *coli*-LPS-stimulated macrophages was reduced by GW-A2 in a dose-dependent manner (Fig 2D).

### GW-A2 exhibited prophylactic and therapeutic effects on LPS-activated macrophages

**Fig 3:**
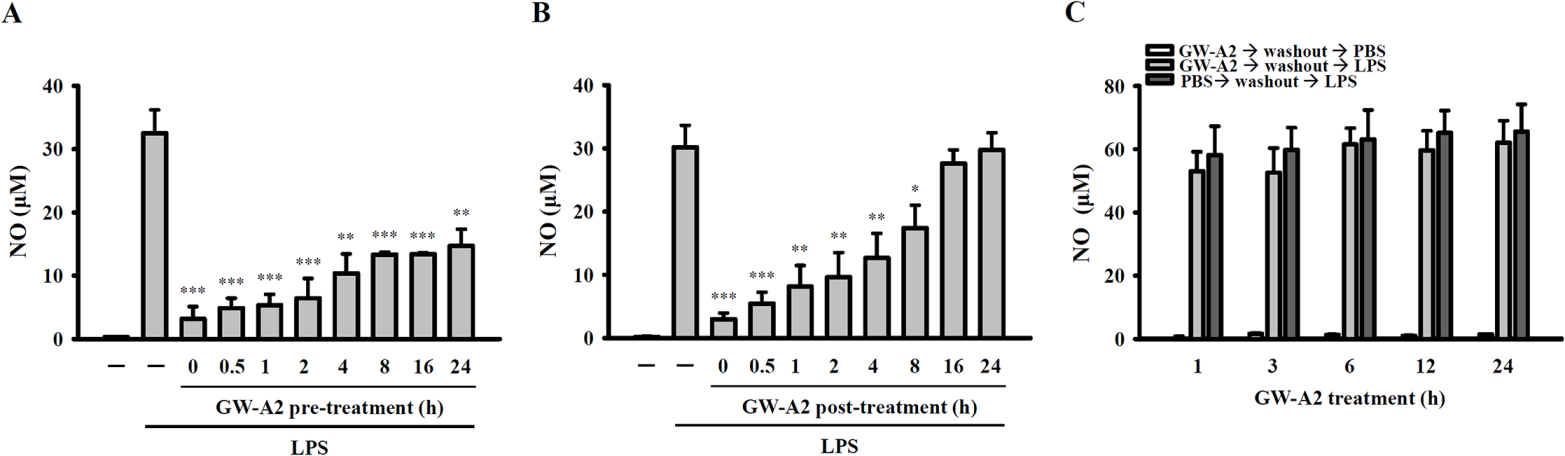
Influence of GW-A2 on the prophylactic and therapeutic effects of LPS-induced NO generation. (**A**) RAW 264.7 macrophages were incubated for 0–24 h with or without GW-A2, followed by 24 h with or without 0.1 μg/ml of *E*. *coli* LPS. The levels of NO in the culture medium were measured by the Griess reaction. (**B**) RAW 264.7 macrophages were incubated for 24 h with or without 0.1 μg/ml of *E*. *coli* LPS, in the presence or absence of 2 μM of GW-A2 in the last 0–24 h. The levels of NO in the culture medium were measured by the Griess reaction. (**C**) RAW 264.7 macrophages were incubated for 0–24 h with or without GW-A2. Then, the cells were washed and the supernatant was replaced with fresh media and stimulated for 24 h with or without 0.1 μg/ml of *E*. *coli* LPS. The levels of NO in the culture medium were measured by the Griess reaction. The data are expressed as the mean ± SD of three independent experiments. *, ** and *** indicate significant differences, representing *p* < 0.05, *p* < 0.01 and *p* < 0.001, respectively compared to LPS alone.

To investigate whether GW-A2 can be used as a prophylactic agent in the treatment of LPS-induced inflammatory response, RAW 264.7 cells were pre-incubated with GW-A2 for 0–24 h, stimulated by LPS for an additional 24 h, and then assessed for NO generation. We found that GW-A2 was able to significantly reduce the generation of LPS-induced NO when GW-A2 was added either with LPS or 24 h before adding LPS (Fig 3A). Moreover, we assessed whether GW-A2 can be used as a therapeutic agent in treating LPS-induced inflammatory response by adding GW-A2 into cell cultures 0–24 h post LPS stimulation. We found that GW-A2 significantly inhibited NO generation when added to cell culture 0–8 h post LPS stimulation, but it had no effect when it was added 16 h after LPS stimulation (Fig 3B). Furthermore, we evaluated whether GW-A2**-**mediated anti-inflammatory effects are caused by the induction of intracellular anti-inflammatory factors. To address this question, the following procedure was conducted. The cells were pre-incubated with GW-A2 or PBS for 1, 3, 6, 12 or 24 h before their supernatant was replaced with fresh media. The cells were then stimulated with LPS for 24 h; following this, we analyzed the generation of NO. We found that GW-A2 pre-treatment was not able to inhibit LPS-induced NO generation when GW-A2 was washed off before the LPS stimulation step (Fig 3C). These results indicated that GW-A2-mediated NO downregulation was not caused by the induction of intracellular anti-inflammatory factors.

### GW-A2 reduces NLRP3 inflammasome-derived IL-1β secretion

**Fig 4:**
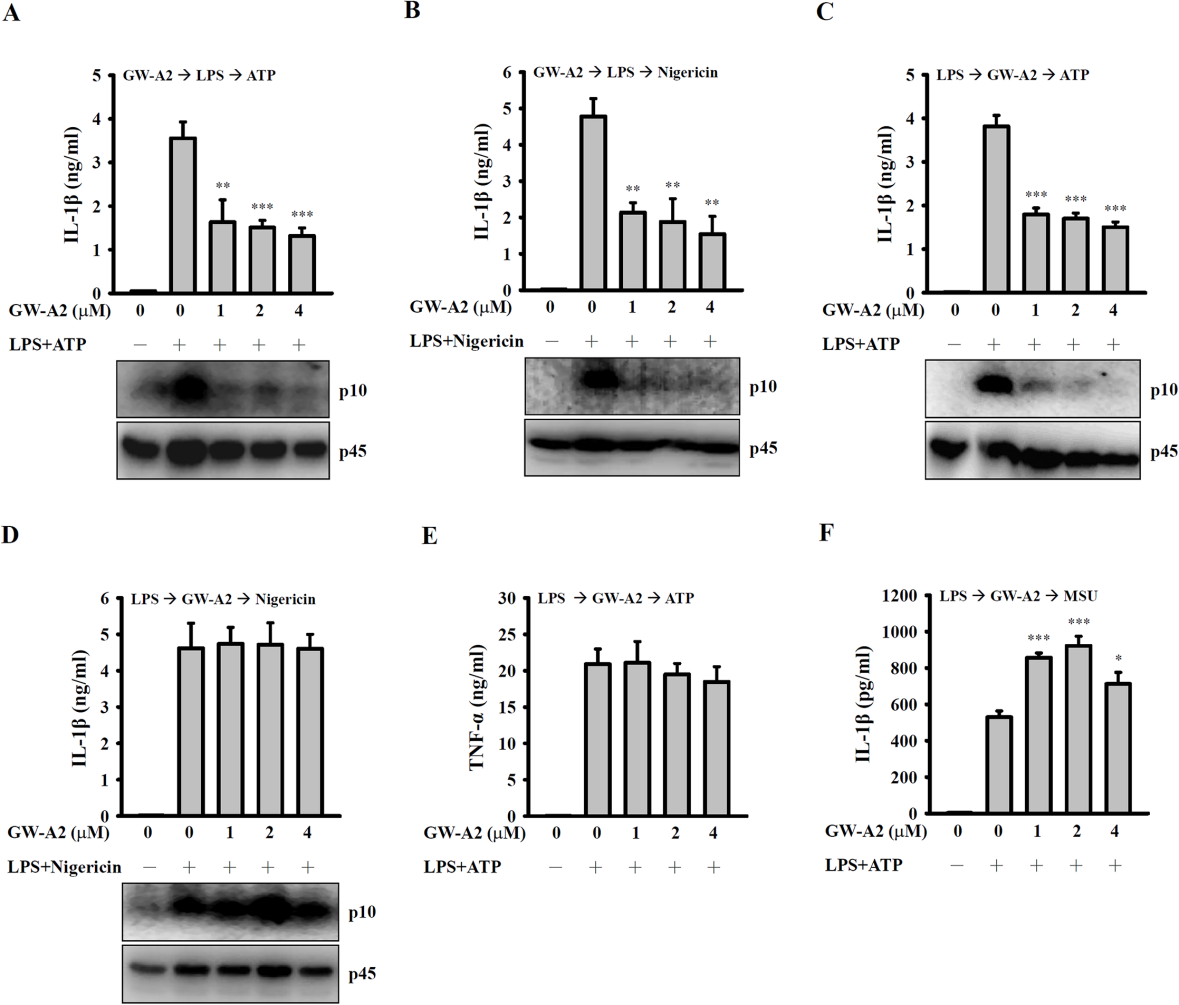
Effects of GW-A2 on NLRP3 inflammasome activation. (**A** and **B**) J774A.1 macrophages were incubated for 30 min with or without 2 μM of GW-A2, for 5.5 h with or without 0.1 μg/ml of *E*. *coli* LPS, and then for 30 min with or without 5 mM of ATP (**A**) or 10 μM of nigericin (**B**). The levels of IL-1β in the culture medium and activated caspase-1 (p10) in the cells were measured by ELISA and Western blotting, respectively. (**C**—**F**) J774A.1 macrophages were incubated for 5.5 h with 0.1 μg/ml of *E*. *coli* LPS, for 30 min with or without 2 μM of GW-A2, and then for 30 min with or without 5 mM of ATP (**C** and **E**), 10 μM of nigericin (**D**) or 100 μg/ml of MSU (**F**). The levels of IL-1β and TNF-α in the culture medium and activated caspase-1 (p10) in the cells were measured by ELISA and Western blotting, respectively. The data are expressed as the mean ± SD of three independent experiments. The Western blotting results shown are a representative experiment of three independent experiments. *, ** and *** indicate significant differences, representing *p* < 0.05, *p* < 0.01 and *p* < 0.001, respectively compared to stimulation alone.

Full activation of the NLRP3 inflammasome requires both a priming signal from LPS-stimulated Toll-like receptor 4 and an activation signal from a second stimulus, such as ATP [17]; the former controls the expression of NLRP3 and proIL-1β and the latter controls caspase-1 activation. Incubation of J774A.1 macrophages with GW-A2 significantly inhibited the IL-1β secretion and caspase-1 activation that was induced by LPS+ATP (Fig 4A) and LPS+nigericin (Fig 4B). These results indicated that GW-A2 was able to inhibit NLRP3 inflammasome activation. We then determined whether GW-A2 influences the priming signal received from LPS or the activation signal received from ATP and nigericin. We incubated J774A.1 macrophages with LPS for 5.5 h (LPS priming), followed by incubation with GW-A2 for 30 min prior to stimulation by ATP or nigericin. We found that GW-A2 inhibited ATP-induced IL-1β secretion and caspase-1 activation (Fig 4C). However, as shown in Fig 4D, induction by nigericin had no significant effect on IL-1β secretion and caspase-1 activation. In addition, under the same conditions, GW-A2 did not decrease TNF-α secretion, which is independent of NLRP3 inflammasome activity (Fig 4E). Furthermore, GW-A2 was not able to inhibit MSU-induced IL-1β secretion in LPS-primed J774A.1 macrophages (Fig 4F). These results indicated that GW-A2 inhibits ATP-mediated, but not nigericin- or MSU-mediated, activation of the NLRP3 inflammasome.

### GW-A2 reduces LPS-induced inflammatory mediator expression in vivo

**Fig 5:**
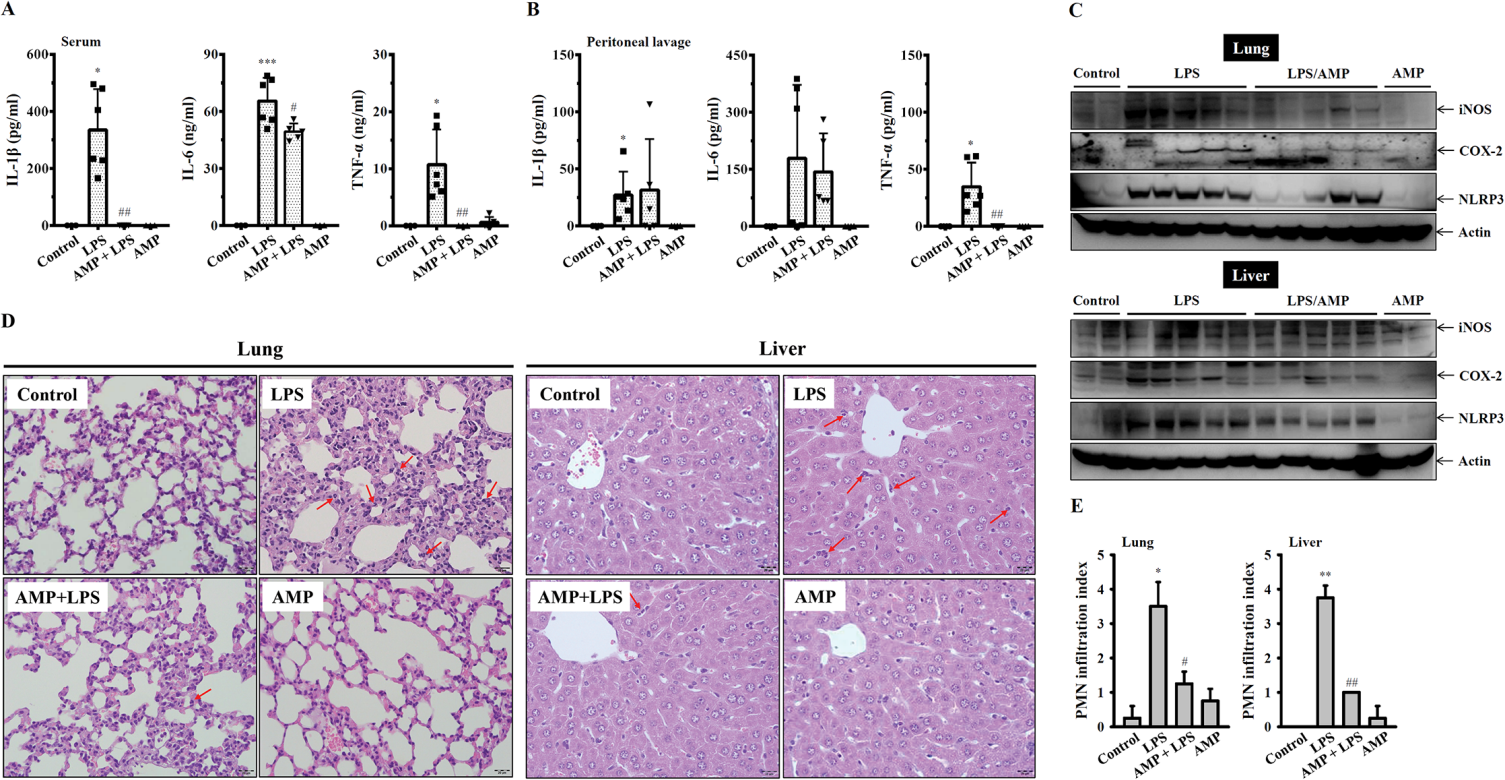
Anti-inflammatory effects of GW-A2 in vivo. (**A**) The levels of IL-1β, IL-6 and TNF-α in the serum collected 4 h after LPS injection were measured by ELISA. (**B**) The levels of IL-1β, IL-6 and TNF-α in the peritoneal lavage collected 24 h after LPS injection were measured by ELISA. (**C**) The levels of iNOS, COX-2 and NLRP3 in lung and liver were assayed by Western blotting. (**D**) The H&E staining of lung and liver tissue sections from the indicated group. The infiltration of PMN was indicated by red arrows. Original magnification x 400. (**E**) PMN infiltration index of lung and liver tissue sections. * and ** indicate significant differences, representing *p* < 0.05 and *p* < 0.01, respectively compared to control mice. # and ## indicate significant differences, representing *p* < 0.05 and *p* < 0.01, respectively compared to LPS-injected mice.

To investigate whether GW-A2 is able to reduce the expression of inflammatory mediators in LPS-injected mice, experimental mice were intraperitoneally injected with LPS or PBS in the presence or absence of GW-A2. Their sera were collected 4 h after LPS injection, and the peritoneal lavages and tissues (lung and liver) were collected 24 h after LPS injection. We found that GW-A2 significantly reduced IL-1β, IL-6 and TNF-α levels in the sera (Fig 5A), but only significantly reduced IL-6 levels in the peritoneal lavages (Fig 5B). The expression levels of iNOS, COX-2 and NLRP3 in the lung and liver of the LPS-injected mice were increased, and these effects were reduced by GW-A2 (Fig 5C). In addition, LPS induced infiltration of PMN into the lung and liver, and these effects were reduced by GW-A2 (Fig 5D and 5E). These results indicated that GW-A2 exhibited anti-inflammatory activity in vivo.

### GW-A2 interacts with LPS and ATP

**Fig 6:**
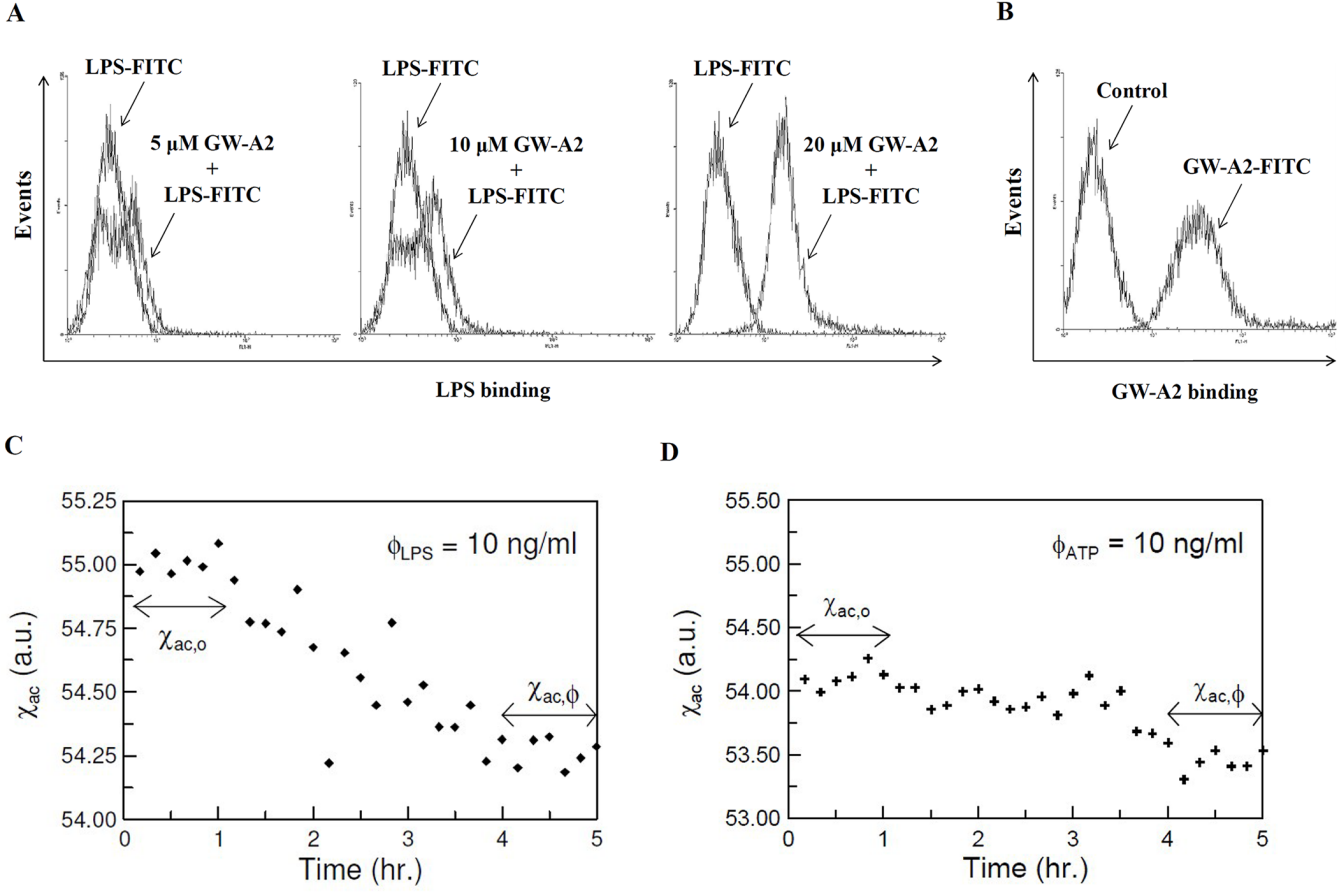
GW-A2 interacts with LPS and ATP. (**A**) Paraformaldehyde-fixed RAW 264.7 macrophages were incubated for 30 min with or without GW-A2 and then for 30 min with or without 2 μg/ml of LPS-FITC at 4°C before being examined by flow cytometry. (**B**) Paraformaldehyde-fixed RAW 264.7 macrophages were incubated for 30 min with or without 20 μM of GW-A2-FITC at 4°C and then examined by flow cytometry. (**C**) Real-time magnetic response, χac, of a mixture of GW-A2-coated magnetic nanoparticles and 10 ng/ml of LPS. (**D**) Real-time magnetic response, χac, of a mixture of GW-A2 coated magnetic nanoparticles and 10 ng/ml of ATP.

GW-A2 significantly inhibits LPS-mediated inflammatory responses. Therefore, we assessed whether GW-A2 affects the binding of LPS to the cell surfaces of macrophages. We found that GW-A2 did not compete with fluorescence-conjugated LPS (LPS-FITC) for cell surface binding; however, an increase in LPS-FITC binding to the cell surface was observed (Fig 6A). In addition, GW-A2 was found to bind to the cell surface, which was shown by a significant increase in the fluorescence emitted by fluorescence-conjugated GW-A2 (GW-A2-FITC)-treated cells (Fig 6B). These results suggested the possibility that GW-A2 binds to LPS and transfers LPS onto the cell surface. To provide direct evidence of GW-A2’s interaction with LPS, an ImmunoMagnetic Reduction (IMR) assay was used [35]. Fig 6C shows a typical example of a real-time magnetic response, χac, of a mixture of magnetic reagent and 10 ng/ml LPS, shown in black dots. The data collected from 0 to 1 h denote the magnetic response, χac,o of the mixture of the magnetic reagent with 10 ng/ml LPS prior to the incubation. The time-average value of χac,o was measured as 55.00. From 1 to 4 h, the χac value showed a declining trend. These results demonstrated that the GW-A2-coated magnetic nanoparticles were reacting with LPS, corresponding to the incubation period. After the reaction/incubation at room temperature (~25°C), the magnetic response, χac,ϕ of the mixture of magnetic reagent and 10 ng/ml LPS solution reached another stable value, as shown in the data collected from 4 to 5 h. The time-average value of χac,ϕ was approximately 54.26. The significant reduction in the magnetic response is evidence of the conjugation between magnetic nanoparticles and LPS. Furthermore, the IMR signal was calculated as IMR (%) = (55.00–54.26)/55.00 x 100% = 1.34%. For the triple tests, the mean value and the standard deviation were 1.33% and 0.01%, respectively. The CV value was 0.75%. In addition, by using this IMR assay, we have demonstrated that GW-A2-coated magnetic nanoparticles reacted with ATP. The time-average values of χac,o and χac, ϕ were 54.09 and 53.46, respectively (Fig 6D). The IMR signal was calculated as IMR (%) = (54.09–53.46)/54.09 x 100% = 1.16%. For the triple tests, the mean value and the standard deviation were 1.15% and 0.021%, respectively. The CV value was 1.83%.

## Discussion

It is well known that AMPs not only help defend against bacteria, viruses, fungi, and parasites but also modulate the immune responses of various organisms [8,9]. It has been reported that AMPs inhibit LPS-induced pro-inflammatory response [22,23,41]. However, the mode of action of these peptides is not yet well understood. Previous investigations showed that the neutralization of LPS by AMPs might be one of the anti-inflammatory actions of AMPs [42–47]. Neutralization of LPS by AMPs may change the type of LPS aggregation from cubic into multilamellar and cause an increase in aggregate size. These changes inhibit the binding of LPS to its receptors and to other mammalian proteins [43]. In addition to the neutralization of LPS, an α-helical cationic AMP inhibits *Propionibacterium acnes*-induced cytokine secretion in primary human keratinocytes, partially by binding to bacterial lipoteichoic acid [48]. In this current study, we demonstrated that the synthetic cationic AMP GW-A2 significantly inhibited LPS-induced inflammatory response, and this effect may involve the binding of GW-A2 to LPS. Although anti-inflammatory AMP may act by blocking LPS binding to target cells [42], GW-A2 was not observed to block the binding of LPS to macrophages; in fact, the binding increased. Our data suggest that the GW-A2/LPS complex binds to macrophages via interactions between GW-A2 and macrophages and not via interaction between LPS and its receptor, TLR-4. Moreover, GAPDH was identified to directly bind to the antimicrobial peptide LL37 in monocytes [49]; however, the cellular receptor for GW-A2 has not yet been identified.

LPS delivers a priming signal to the NLRP3 inflammasome, which is essential for its activation [50]. A secondary signal induces formation of the NLRP3 inflammasome complex, which induces the processing of pro-IL-1β by activated caspase-1. Complex formation and activation can be triggered by different stimuli, including bacterial toxins, endogenously expressed sterile stimuli (uric acid and cholesterol crystals etc.), and necrotic cells [51]. ATP produced by mitochondria and released by necrotic cells triggers a sterile inflammatory response through the NLRP3 inflammasome [52]. Hilpert et al. showed that certain cationic AMPs interact with ATP and directly inhibit the activities of ATP-dependent enzymes. These effects were highly dependent on whether the peptide structure was α-helical and planar; circular AMPs and other cationic peptides, including polymyxin B and gramicidin, failed to inhibit ATP-dependent enzymes [53]. Although cationic GW-A2 binds to negatively charged LPS and ATP, inhibiting their induction of inflammatory responses, these effects are not completely dependent on charge effects: scrambled GW-A2 has the same charge as unmodified GW-A2, but it failed to inhibit LPS-induced NO generation. In addition, GW-A2 inhibits NO generation that is induced by positively charged Pam3CSK4 [54]. These results indicated that the anti-inflammatory activity of GW-A2 is dependent on peptide structure. In particular, GW-A2 not only inhibits LPS-mediated response but also inhibits ATP-mediated caspase-1 activation and IL-1β secretion, suggesting that cationic AMPs can potentially be used to inhibit the NLRP3 inflammasome [24].

Based on the chemical properties of GW-A2, it is a positively charged molecule. We assume that GW-A2 can bind to negatively charged molecules such as LPS and ATP. Based on our data shown in Fig 6D and 6E, GW-A2 binds to ATP and LPS directly. We hypothesize that binding of LPS and ATP to GW-A2 may affect the interaction between LPS and TLR4 as well as ATP and P2X7 receptor, and leading to the impaired downstream responses. The hypothesis was partially evidenced as GW-A2 inhibited ATP-mediated IL-1β secretion and caspase-1 activation in LPS-primed macrophages (Fig 4C), but had no effect on nigericin- and MSU-mediated IL-1β secretion and caspase-1 activation (Fig 4D and 4F), because nigericin and MSU are not negatively charged molecules. In addition, the phosphorylation levels of MAPK, PKC-α and PKC-δ in LPS-activated macrophages peak at 10–20 min and then gradually disappear (Fig 2A and 2B). However, GW-A2 worked still very well to inhibit LPS-induced NO production even after post-treatment for 8 h (Fig 3B). These results suggested that GW-A2 inhibit LPS-induced NO production partially depends on MAPK, PKC-α and PKC-δ associated pathways, and there should be some unidentified pathways inhibited by GW-A2.

AMPs control infections not only by killing bacteria but also by reducing inflammation via neutralizing pathogen-associated molecular patterns [3,24,53]. Although AMPs have a broad spectrum of anti-microbial activities, low levels of induced resistance, and broad anti-inflammatory activities [55], no AMPs have been approved by the Food & Drug Administration for the treatment of topical or systemic medical indications [55,56]. AMPs have not passed clinical trials due to their high levels of toxicity, which causes hemolysis, nephrotoxicity and neurotoxicity [55]. The economic viability of AMPs is limited because a number of naturally occurring AMPs have been patented. Synthesizing peptides based on naturally occurring forms of AMPs might overcome patent exclusivity. In this study we demonstrated that the synthetic cationic antimicrobial peptide GW-A2 was able to inhibit inflammatory response and the NLRP3 inflammasome. Compared to the commercialized small molecules used to inhibit LPS and NLRP3 pathways, one of the advantages of GW-A2 is that it not only shows antimicrobial activities [25,26] but also concomitant the anti-inflammatory activities. Another potentially advantage of GW-A2 is the low levels of induced drug resistance [57]. However, before the GW-A2 can be used in the clinic applications, there are some disadvantages should be taken into consideration. One is the high synthesis and manufacturing costs compared to the small molecules. In addition, although the behaviors of control mice and GW-A2-injected mice were not significantly different and no adverse effects were observed in GW-A2-injected mice, the potential systemic and local toxicity and the allergy after repeated application and long term usage should be further investigated [58].
